# Intraspecific phytochemical variation shapes community and population structure for specialist caterpillars

**DOI:** 10.1111/nph.14038

**Published:** 2016-06-09

**Authors:** Andrea E. Glassmire, Christopher S. Jeffrey, Matthew L. Forister, Thomas L. Parchman, Chris C. Nice, Joshua P. Jahner, Joseph S. Wilson, Thomas R. Walla, Lora A. Richards, Angela M. Smilanich, Michael D. Leonard, Colin R. Morrison, Wilmer Simbaña, Luis A. Salagaje, Craig D. Dodson, Jim S. Miller, Eric J. Tepe, Santiago Villamarin‐Cortez, Lee A. Dyer

**Affiliations:** ^1^ Ecology, Evolution, and Conservation BiologyUniversity of Nevada1664 N. Virginia StRenoNV89557USA; ^2^ Department of ChemistryUniversity of Nevada1664 N. Virginia StRenoNV89557USA; ^3^ Department of BiologyTexas State University601 University Dr.San MarcosTX78666USA; ^4^ Department of BiologyUtah State University Tooele1021 W Vine StTooleUT84074USA; ^5^ Department of BiologyColorado Mesa University1100 N. AveGrand JunctionCO81501USA; ^6^ Museo Ecuatoriano de Ciencias Naturales del Instituto Nacional de Biodiversidad EcuadorRumipamba 341 y Av. Shyris.QuitoEcuador; ^7^ Yanayacu Biological StationCosangaNapo ProvinceEcuador; ^8^ Department of Biological SciencesUniversity of Cincinnati318 College DrCincinnatiOH45221USA

**Keywords:** chemical interactions, community structure, *Eois*, multi‐trophic, phytochemical variation, *Piper*, population diversification

## Abstract

- Chemically mediated plant–herbivore interactions contribute to the diversity of terrestrial communities and the diversification of plants and insects. While our understanding of the processes affecting community structure and evolutionary diversification has grown, few studies have investigated how trait variation shapes genetic and species diversity simultaneously in a tropical ecosystem.
- We investigated secondary metabolite variation among subpopulations of a single plant species, *Piper kelleyi* (Piperaceae), using high‐performance liquid chromatography (HPLC), to understand associations between plant phytochemistry and host‐specialized caterpillars in the genus *Eois* (Geometridae: Larentiinae) and associated parasitoid wasps and flies. In addition, we used a genotyping‐by‐sequencing approach to examine the genetic structure of one abundant caterpillar species, *Eois encina*, in relation to host phytochemical variation.
- We found substantive concentration differences among three major secondary metabolites, and these differences in chemistry predicted caterpillar and parasitoid community structure among host plant populations. Furthermore, *E. encina* populations located at high elevations were genetically different from other populations. They fed on plants containing high concentrations of prenylated benzoic acid.
- Thus, phytochemistry potentially shapes caterpillar and wasp community composition and geographic variation in species interactions, both of which can contribute to diversification of plants and insects.

## Abstract 2

See also the Commentary on this article by Kessler, **212**: 8–10.

## Introduction

Chemically mediated interactions between plants, herbivores and natural enemies have important ecological and evolutionary impacts on biodiversity (Ehrlich & Raven, 1964; Becerra *et al*., 2009; Wilson *et al*., 2012). Despite considerable progress in understanding relationships between trophic interactions and diversity, a knowledge gap remains: specifically, how does phytochemical diversity affect multi‐trophic interactions at community and population levels? There has been progress towards addressing these questions using clonal plants to experimentally manipulate genetic diversity and quantify associated increases in arthropod richness (Crutsinger *et al*., 2006; Johnson *et al*., 2006). However, there are limitations to using clones or similar experimental approaches for understanding natural variation, especially in mega‐diverse systems; these approaches do not accurately reflect levels of interaction diversity and genetic diversity observed in nature (reviewed in Whitham *et al*., 2006; Hughes *et al*., 2008; Crutsinger, 2015). Furthermore, manipulative experiments examining multi‐trophic interactions have focused on the unidirectional influence of plant genetic diversity on arthropod richness (Fritz & Price, 1988; Johnson & Agrawal, 2005; Whitham *et al*., 2006), whereas only a few studies have quantified genetic and species diversity of herbivores simultaneously (Fridley & Grime, 2010; Crawford & Rudgers, 2013; Abdala‐Roberts *et al*., 2015). Most of these experimental examples have been limited to temperate grasslands and forests dominated by a foundation species (reviewed in Whitham *et al*., 2006; Hughes *et al*., 2008; Crutsinger, 2015), as opposed to examining these relationships in highly diverse ecosystems that lack a single dominant species. Here we focused on a model plant–herbivore–parasitoid system to examine effects of plant chemical diversity on community‐ and population‐level processes in highly diverse tropical forests.

The links between plant genetic diversity and the diversity of upper trophic levels are partially mediated via changes in phytochemical diversity (Richards *et al*., 2015). Although we currently lack an understanding of how population genetic and genomic variation predict phytochemical diversity, secondary metabolite concentrations are heritable phenotypes (Geber & Griffen, 2003; Johnson *et al*., 2009; Barbour *et al*., 2015) that can respond to selection and can directly and indirectly impact biotic communities (Bailey *et al*., 2006) and ecosystems (Driebe & Whitham, 2000; Whitham *et al*., 2003; Schweitzer *et al*., 2005). Intraspecific chemical variation between individual host plants could give rise to geographically variable selection, and contribute to shifts in herbivore preference for, or performance on, unique concentrations of individual secondary compounds (i.e. a selection mosaic; Thompson, 1999, 2005). This geographic variation in turn could affect the make‐up of herbivore and predator assemblages and might allow for greater species packing, contributing to higher alpha diversity (Rodríguez‐Castañeda *et al*., 2010; Richards *et al*., 2015). Furthermore, geographically separated herbivore populations may adapt locally to a specific chemical profile in intraspecific host plants, and this could reduce effective migration between habitats (Wilson *et al*., 2012). Thus, phytochemical variation across host plant populations might shape the composition of herbivore assemblages, and could give rise to geographically divergent selection on herbivore populations.

To examine such relationships, we focused on a well‐studied tropical genus of caterpillars, *Eois* (Geometridae), that feed exclusively on plants in the genus *Piper* (Piperaceae) and are host to diverse parasitoid communities (Dyer & Palmer, 2004). As recently as the Pleistocene, multiple, independent clades of *Eois* caterpillars have undergone geographically localized radiations, in some cases with sister species of *Eois* in the same geographic area, associated with the same host plant species (Wilson *et al*., 2012). This pattern of sympatric sister species utilizing the same resource makes it difficult to assume a framework of ecological divergence in allopatry associated with shifting host species. However, intraspecific variation in plant chemical defense could contribute to divergence (Wilson *et al*., 2012). *Piper* is chemically defended by a remarkable diversity of secondary compounds that exhibit a broad array of biological activities. These include antibacterial (Diaz *et al*., 2012), antitubercular (Diaz *et al*., 2012), antifungal (Johann *et al*., 2009), and anti‐herbivore effects (Dyer *et al*., 2003; Jeffrey *et al*., 2014). *Piper* chemical defenses are known to vary within as well as among species and have been found to function additively or synergistically to deter or poison herbivores (Dyer *et al*., 2003; Richards *et al*., 2010) and could decrease the herbivores' defenses against parasitoids (Smilanich *et al*., 2009). Considering the highly diverse chemistry of *Piper*, it is possible that sufficient intraspecific variation exists to facilitate divergence at small spatial scales. Thus, different populations of host species might provide sufficiently different niches to affect the evolution of herbivore and parasitoid communities and to facilitate local herbivore divergence.

To investigate intraspecific phytochemical variation and the consequences for both communities and populations of herbivores, we collected specialist *Eois* caterpillars and the leaves of *Piper kelleyi* Tepe they were consuming across an elevational gradient. Our goals were to understand how the abundance and composition of plant secondary metabolites within localized host plant populations might influence caterpillar and parasitoid communities along an elevational gradient, and how variation among host plant populations might influence genetic variation of one *Eois* species. We tested the following hypotheses: *P. kelleyi* populations are characterized by predictable variation in phytochemistry along an elevational gradient; phytochemical variation is a driver of caterpillar and parasitoid community structure across different microhabitats containing *P. kelleyi*; phytochemical variation is associated with population genetic variation in the most common *Eois* species.

## Materials and Methods

### Study system and data collection

**Figure 1:**
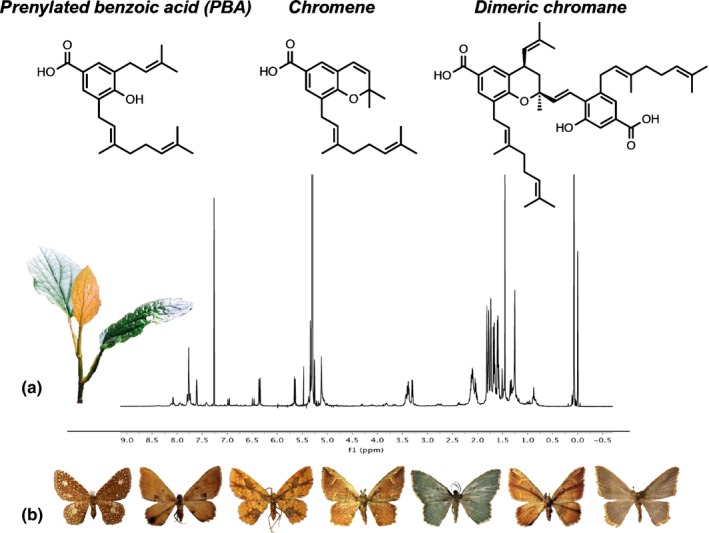
*Piper kelleyi* leaf chemistry and *Eois* caterpillar community study system. (a) NMR spectra of the crude extract containing the three major secondary compounds that have been isolated from the leaves of *P. kelleyi*; a specific prenylated benzoic acid, chromene and dimeric chromane. (b) Species of *Eois* that specialize on *P. kelleyi* include (from left to right): *Eois planetaria* Dognin, *Eois* aff*. encina* Dognin, *Eois ignefumata* Dognin, *Eois encina* Dognin, *Eois viridiflava* Dognin, *Eois* aff*. pallidicosta* Warren, and *Eois* aff. *viridiflava* Dognin.

*Piper kelleyi* Tepe is endemic to the eastern slopes of the Andes of Ecuador and Peru, occurring within a narrow altitudinal range between 1400 and 2400 m (Tepe *et al*., 2014). It is a mid‐canopy shrub characterized by broad ovate leaves and is commonly called ‘pink belly’ because of the distinct pink coloration on the ventral surface of younger leaves (Tepe *et al*., 2014). *Piper kelleyi* hosts an unusually high diversity of specialist caterpillars from the genus *Eois* which in turn are hosts to several species of parasitoid wasps and flies (Tepe *et al*., 2014). The major secondary compounds in the leaves of this species are a specific prenylated benzoic acid, chromene and dimeric chromane, which make up > 95% of the compounds present in the crude extract and are present at a high concentration of ~10% of the dry weight of the leaf material (Fig. 1a), and which all have significant negative effects on herbivores, microbes and fungi (Jeffrey *et al*., 2014). We collected *P. kelleyi*,* Eois* caterpillars and parasitoids from 10 sites in close proximity to Yanayacu Biological Station (00°36′S and 77°53′W) from June to August 2011 and 2012. Yanayacu, comprising disturbed habitat combined with pristine cloud forest, is located in the eastern Andes (Napo Province, Ecuador). The 10 sites were separated by elevation and consisted of four replicated plots that were randomly selected within sites. The temporary plots were 10 m in diameter. Every *P. kelleyi* plant was sampled for *Eois* caterpillars within these plots; the number of *P. kelleyi* plants in each plot was variable. The overall sampling resulted in a total of 125 plants and 2318 caterpillars across 40 plots. Eight of the plots were excluded because of missing leaf samples or no caterpillars being found (Supporting Information Fig. S1). Caterpillars were reared to adult moths or parasitoids to identify species and to calculate levels of parasitism. Caterpillars were identified to species level (Fig. 1b; *Eois encina* Dognin, *Eois* aff*. encina* Dognin, *Eois ignefumata* Dognin, *Eois* aff. *pallidicosta* Warren, *Eois planetaria* Dognin, *Eois viridiflava* Dognin, and *Eois* aff. *viridiflava* Dognin) based on photographs that were taken at every instar. Larval and adult images were compared to image vouchers (http://www.caterpillars.org; Dyer *et al*., 2010) and museum vouchers to assess species identifications. Genitalia were dissected from adult moths to confirm species determinations. Plant material was identified, and voucher specimens (Tepe & Moreno 2999 MO, QCA, QCNE; Glassmire B13 CINC, QCNE) were deposited at the Herbario Nacional del Ecuador, Quito, Ecuador (QCNE), the herbarium of the Pontificia Universidad Católica del Ecuador (QCA), the herbarium at the University of Cincinnati (CINC), and the Missouri Botanical Garden, USA (MO). In addition to *P. kelleyi* sampling, we sampled plant richness and total leaf abundances for all other *Piper* species in each plot. All collections were conducted with permission from the Museo Ecuatoriano de Ciencias Naturales in Quito, Ecuador (permit no. 001‐2011‐DPAP‐MA).

### HPLC analysis of *P. kelleyi* leaves

Young leaves were collected from 93 of the *P. kelleyi* shrubs that were sampled for caterpillars to quantify correlations between plant chemistry and caterpillar genetics. The leaves were dried at 25°C in a dry box at the field station. The compounds are thermally stable and incident light over relatively short periods of time is not known to cause decomposition (Jeffrey *et al*., 2014). We stored samples in a dark freezer to reduce light exposure during the extraction and analysis process. In the laboratory, individual leaves were ground using liquid nitrogen, mortar and pestle, and then 250 mg of leaf material was extracted with 2 ml of high‐performance liquid chromatography (HPLC)‐grade methanol for each leaf (full methods with justifications are provided in Jeffrey *et al*., 2014). The extract was sonicated for 15 min and the insoluble leaf material was removed by vacuum filtration. This entire extraction protocol was repeated twice. The methanol was removed under reduced pressure using rotary evaporation and placed under a high vacuum for 24 h, to remove residual solvent. The remaining crude extract was dissolved in HPLC‐grade methanol with an internal standard of 0.3 mg ml^−1^ methyl salicylate (Sigma‐Aldrich; product number M6752). Samples were analyzed by HPLC using a Phenomenex Luna C18 (Phenomenex Co., Torrance, CA, USA) reverse phase column (150 × 4.6 mm, 5 micron) and an Agilent Technologies (Santa Clara, CA, USA) 1200 series instrument coupled to a diode array detector (DAD) detecting at 280 nm. The solvent system employed was HPLC‐grade methanol with 0.01% trifluoroacetic acid (TFA; Sigma‐Aldrich; product no. T62200) and HPLC‐grade water with 0.01% TFA. The 5‐μl injection was eluted at a constant flow of 1 ml min^−1^ with a gradient of methanol and water as follows: 0–15 min, 50–100% methanol; 15–30 min, 100% methanol; 30–31 min, 100–50% methanol; 31–36 min, 50% methanol. The internal standard (methyl salicylate) was observed at retention time *R*_t_ = 8.464 min. The prenylated benzoic acid was observed at *R*_t_ = 6.342 min, the chromene at *R*_t_ = 7.995 min, and the dimeric chromane at *R*_t_ = 8.997 min relative to the internal standard. The relative abundances of prenylated benzoic acid, chromene, and dimeric chromane were quantified using the ratio of peak areas to the internal standard (methyl salicylate). The HPLC response units for area under the peak are given in mAU·s (milliabsorbance units·s).

### Community analyses

We used structural equation models (Shipley, 2002) to test the hypothesis that phytochemical variation is a primary driver of *Eois* caterpillar assemblages based on the locations of *P. kelleyi*, the make‐up of the surrounding *Piper* community, and the diversity of parasitoids. Our focus was on *P. kelleyi*, but we included *Piper* community diversity because it is likely that some of the variance in caterpillar and parasitoid community composition could be attributable to the biology and chemistry of these other plant species (Tahvanainen & Root, 1972; Root, 1973; Barbosa *et al*., 2009). The community data were collected from 32 plots as described in the section ‘Study system and data collection’. From these plots, we collected 1481 *Eois* caterpillars that were described to species level (seven species in total), with 75% of the individuals being *E. encina*. We reared out 280 parasitoids that were identified to family level (Braconidae, Tachinidae, Ichneumonidae and Eulophidae). We quantified the secondary metabolites from 93 *P. kelleyi* plants (see the section ‘HPLC analysis of *P. kelleyi* leaves’). Finally, we measured richness and abundance in the plots for the 12 species of *Piper* plants that were found (*Piper augustum* Rudge, *Piper* sp. 1 ‘cafecito’, *Piper crassinervium* Kunth, *Piper baezanum* Sodiro ex C.DC., *Piper ecuadorense* Sodiro, *Piper hispidum* Sw., *Piper lanceifolium* Kunth, *Piper napo‐pastazanum* Trel. & Yunck., *Piper perareolatum* C.DC., *Piper pubinervulum* C.DC., *Piper schuppii* A.H.Gentry and *Piper silvivagum* C.DC.).

The relative abundances of prenylated benzoic acid, chromene, and dimeric chromane derived in relation to an internal standard from the HPLC were quantified for each individual *P. kelleyi* plant from which *Eois* caterpillars were collected. Concentrations of prenylated benzoic acid, chromene and dimeric chromane were highly correlated using Spearman rank correlations (chromene and dimeric chromane ρ *= *0.91; *P *<* *0.001; dimeric chromane and benzoic acid ρ *= *0.71; *P *<* *0.001; chromene and the benzoic acid ρ *=* 0.59; *P *<* *0.001). As these compounds are hypothesized to be biosynthetically linked, a factor analysis with varimax rotation was used to create a latent variable of phytochemical defense based on shared variance of the relative abundances of each compound. As there were dominant and rare species, we used the inverse of Simpson's diversity index to calculate community entropies and species equivalents (Jost, 2006) for all *Eois* and parasitoid species collected from each *P. kelleyi* plant, and from the other *Piper* species located in *P. kelleyi* plots. Elevation was measured using GPS for each *P. kelleyi* plot.

For our *a priori* specified structural equation model, we included specific causal relationships resulting in a model with one exogenous variable (elevation) predicting three endogenous variables (*Eois* diversity per plant, parasitoid diversity per plant, and *Piper* diversity per plot); the phytochemical defense factor (from the aforementioned factor analysis) was included as a latent variable. These five variables were included in our model with hypothesized relationships (Table 1) that are context dependent and based on previous work with *Piper*,* Eois*, and parasitoids (Dyer *et al*., 2003, 2004; Brehm *et al*., 2007; Connahs *et al*., 2009; Smilanich *et al*., 2009; Rodríguez‐Castañeda *et al*., 2010; Wilson *et al*., 2012; Richards *et al*., 2015). For example, it was hypothesized that higher relative concentrations of secondary metabolites will decrease the diversity of specialist herbivores as a consequence of higher levels of toxicity (Poelman *et al*., 2009; Richards *et al*., 2015), and this will positively affect parasitism, because sequestered toxins impair the caterpillars' immune response when consumed (Smilanich *et al*., 2009; Richards *et al*., 2012).

**Table 1:** Hypotheses and *a priori* predictions guiding the structural equation model

Predictor variable	Response variable	Causal relationship	Hypothesis and prediction	Citations
*Piper kelleyi* phytochemical defense	*Eois* community diversity	I	The screening hypothesis posits that plants with a higher diversity of secondary metabolites have a greater probability of being toxic to a broad array of herbivores, as a result of unique mixtures and synergies that deter plant enemies. This yields the prediction that the diversity of *P. kelleyi* secondary metabolites will decrease *Eois* herbivore diversity through diverse defensive mechanisms.	Poelman *et al*. (2009); Smilanich *et al*. (2009); Firn (2010); Richards *et al*. (2012, 2015)
*Piper* community diversity	*Piper kelleyi* phytochemical defense	II	According to the associational resistance hypothesis, different plant species occurring in close proximity can decrease the likelihood of detection by herbivores. If diversity of *Piper* shrubs is high, then it will be harder for herbivores to detect *P. kelleyi* (or other species). Thus, *P. kelleyi* may invest less in producing defensive compounds in high *Piper* diversity communities because of low vulnerability to herbivores.	Root (1973); Tahvanainen & Root (1972); Barbosa *et al*. (2009)
*Piper kelleyi* phytochemical defense	Parasitoid community diversity	III	Plants interact with parasitoids by providing chemical cues for defense against herbivores or poisoning the immune response of caterpillars. If phytochemical defense increases, then parasitoid community diversity should increase.	Turlings & Ton (2006); Smilanich *et al*. (2009); Richards *et al*. (2012); Wäschke *et al*. (2014)
Elevation	*Piper kelleyi* phytochemical defense	IV	Phototoxicity occurs for many secondary metabolites when they are exposed to UV light and are metabolized to more toxic compounds or generate reactive intermediates that interfere with DNA or proteins. If UV light intensity increases with increasing elevation, then plants containing secondary metabolites that are photoactive should have a selective advantage at higher elevations.	Downum *et al*. (1991); Krause *et al*. (1999); Ruhland *et al*. (2013); Virjamo *et al*. (2014)
Elevation	*Eois* community diversity	V	Caterpillar diversity should increase as elevation increases, as a result of higher levels of specialization and lower levels of predation at higher elevations.	Brehm *et al*. (2007); Rodríguez‐Castañeda *et al*. (2010)
Elevation	*Piper* community diversity	VI	Plant diversity should decrease as elevation increases (beyond mid‐elevations) as a consequence of a variety of mechanisms, including colder temperatures, lower productivity, and smaller area.	Brehm *et al*. (2007); Rodríguez‐Castañeda *et al*. (2010)
Elevation	Parasitoid community diversity	VII	If herbivore specialization increases at higher elevations, then levels of parasitism and parasitoid diversity are predicted to increase as a result of preferential parasitism of specialists and higher diversities of herbivores. Increased concentrations of secondary metabolites at higher elevations may disrupt the herbivore immune response against parasitoids.	Smilanich *et al*. (2009); Rodríguez‐Castañeda *et al*. (2010); Richards *et al*. (2012)
*Piper* community diversity	*Eois* community diversity	VIII	More complex and diverse plant communities can decrease the abundance of specialist herbivores. This is because diverse plant communities decrease the detectability of preferred host plants for specialist herbivores.	Root (1973); Tahvanainen & Root (1972); Barbosa *et al*. (2009)
*Piper* community diversity	Parasitoid community diversity	IX	Increases in plant diversity can cause increases in parasitoid diversity by providing more host species and stronger signals for host searching and oviposition cues. Parasitoids can discriminate between their host odors and the complex odors produced by the plant community. This appears as a ‘direct’ effect because we have not measured cues or other important determinants of parasitoid diversity.	Erb *et al*. (2010); Wäschke *et al*. (2014)

**Figure 2:**
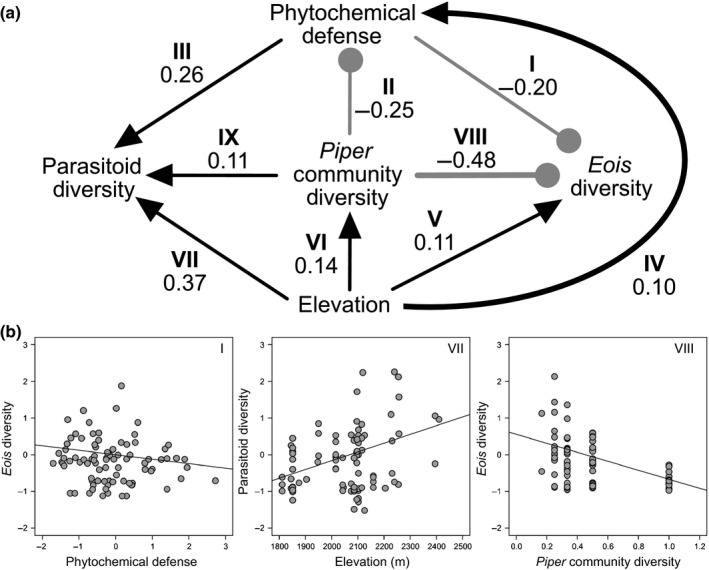
Results of a structural equation model depicting hypothesized causal relationships between: phytochemical defense (latent variable), *Eois* diversity per *P. kelleyi* plant, parasitoid diversity per *P. kelleyi* plant, *Piper* species diversity per plot, and elevation. (a) Illustration of the overall path model. The direct positive effects are indicated by black arrows, while the direct negative effects are indicated by light gray blunt‐ended lines. The numbers beside the lines are the standardized path coefficients. The Roman numerals above the path coefficients relate to Table 1, which describes specific hypotheses being tested. *Piper kelleyi* phytochemical variation is a latent variable, created via factor analysis on relative abundances of the three defensive compounds with varimax rotation. The path coefficients are all significant (*P *<* *0.05) and the model is a significant fit to the data (χ^2^ = 0.014; df = 1; *P *>* *0.1). (b) A subset of the partial correlation plots from paths I, VII and VIII; the remaining partial correlation plots can be found in Supporting Information Fig. S2.

Roman numerals refer to the causal relationship depicted in the path diagram (Fig. 2). These hypotheses are context dependent and based on previous work in the *Piper*,* Eois*, parasitoid system.

We tested the fit of this model using previously established approaches (Greeney *et al*., 2015) and selected the formulation of the reticular action model to define alternative models. Starting values for the parameter estimates were determined by using a combination of three methods: observed moments of variables, the McDonald method, and two‐stage least squares. The estimation method for the model was maximum likelihood, and the Levenberg−Marquardt algorithm was used to iterate solutions for optimization. The χ^2^ for the absolute index was used to assess the fit of the model, with *P* >** **0.1 (with 4 df) as an indication of a good fit to the data. Residuals met assumptions for the general linear model (i.e. generalized linear model with Gaussian distribution, identity link function, and fixed effects). These analyses were conducted using sas (v.9.1, proc calis; SAS Institute, Cary, NC, USA).

### Caterpillar genetic variation, phytochemical variation, and elevation

We used a genotyping‐by‐sequencing (GBS) approach to generate population genetic data for *E. encina*. DNA was extracted from 155 individual *E. encina* caterpillars. DNA extractions were performed on thoraxes of dry adult specimens using Qiagen DNeasy Blood and Tissue kits (Qiagen Inc., Valencia, CA) and quantified using spectrophotometry. Reduced‐representation genomic libraries for Illumina (Illumina Biotechnology Co., San Diego, CA, USA) sequencing were constructed using a GBS approach (Gompert *et al*., 2012; Parchman *et al*., 2013). Genomic DNA was cut with two restriction enzymes, *Eco*RI and *Mse*I, and a unique DNA barcode was ligated to the fragments from each individual to allow multiplexing. DNA fragment libraries were PCR amplified using standard Illumina primers and size selected for a region between 200 and 300 bases using QIAquick gel extraction kits (Qiagen). One lane of sequencing on the Illumina HiSeq at the National Center for Genome Resources (Santa Fe, NM) generated 47 million 100‐bp reads.

We used a custom Perl script to parse barcodes from sequences and to assign the correct individual ID to each read. As a reference genome is not available for *E. encina*, we used a two‐step assembly procedure to assemble reads into homologous genetic regions. We first performed a *de novo* assembly for a subset of 30 million sequences with seqman ngen 3.0.4 (dnastar, Dnastar Software Co., Madison, WI, USA), using a minimum match percentage of 92%, a gap penalty of 50, and a match size of 50 bp (full details of assembly parameterization are available upon request). We removed low‐quality contigs and sequences < 82 or > 88 bases in length to generate an artificial reference containing 481 606 contig consensus sequences. We then assembled all reads onto the reference using the aln and samse algorithms in bwa (Li & Durbin, 2009), using an edit distance of 3 (full parameters for bwa assemblies are available upon request). We used samtools and bcftools (Li *et al*., 2009) to identify single nucleotide polymorphisms (SNPs) in assemblies and retained SNPs where at least one read was aligned in at least 50% of the individuals. Genotype likelihoods were calculated with bcftools (Li *et al*., 2009), stored in variant call format (VCF), and converted to composite genotype likelihood point estimates for downstream analyses. We summarized genetic variation using principal components (PC) analysis of the genotype covariance matrix using the prcomp package in R (R Development Core Team, 2013).

We created matrices of genetic distances among caterpillar individuals using the calculated PC scores of genotype likelihoods. We created three different matrices based on: (1) all the PCs for an overall genetic representation; (2) PC1 and 2 because they explain most of the variation for population structure; and (3) just PC2 because it distinguishes the higher elevation individuals. We used a multiple regression on distance matrices (MRM; Lichstein, 2007), using the ‘mrm’ function from the ecodist package in R (Goslee & Urban, 2007), with 100 000 permutations. We conducted three models that differed in the response variable. The response variable was the genetic distances based on Euclidean distances for: (1) all the PCs, (2) PC1 and 2, and (3) only PC2, as already described. The independent variables were elevation, individual compound concentrations (i.e. prenylated benzoic acid, chromene, and dimeric chromane) and GPS location from each plant sampled, transformed into separate Euclidean distance matrices.

## Results

### Plant chemistry analyses

We found that prenylated benzoic acid, chromene, and dimeric chromane were present in *P. kelleyi* at a high concentration of ~10% of the dry weight of leaf material (Fig. 1a), which is consistent with previous results (Jeffrey *et al*., 2014). While these compounds were highly correlated and hypothesized to be biosynthetically linked, the relative abundance of each compound can be important for synergistic or additive effects on herbivores (Dyer *et al*., 2003; Richards *et al*., 2010). Thus, we calculated a Shannon equivalence variable (Jost, 2006) for the three compounds, which accurately captures their relative abundances in an individual plant and does not give added weight to one dominant compound as all compounds are present (i.e. the richness of compounds does not change). This compound equivalence variable was used in the structural equation models already described; higher values indicate greater abundance and evenness of the compounds.

### Community analyses

The factor analysis included concentrations of the three different secondary metabolites, utilized varimax rotation, and yielded two factors. Kaiser's measure of sampling adequacy, which assesses the adequacy of correlation matrices for factor analysis, was acceptable (0.6) and the matrix was not an identity matrix. The relative concentrations of all three compounds loaded onto Factor 1 with high loadings for dimeric chromane (0.96) and chromene (0.92) and lower loadings for prenylated benzoic acid (0.73). Factor 2 had more useful loadings for separating prenylated benzoic acid (0.68) from dimeric chromane (−0.18) and chromene (−0.36), and it was utilized in path models as a latent ‘phytochemical defense’ (benzoic acid vs other compounds) variable.

**Figure 3:**
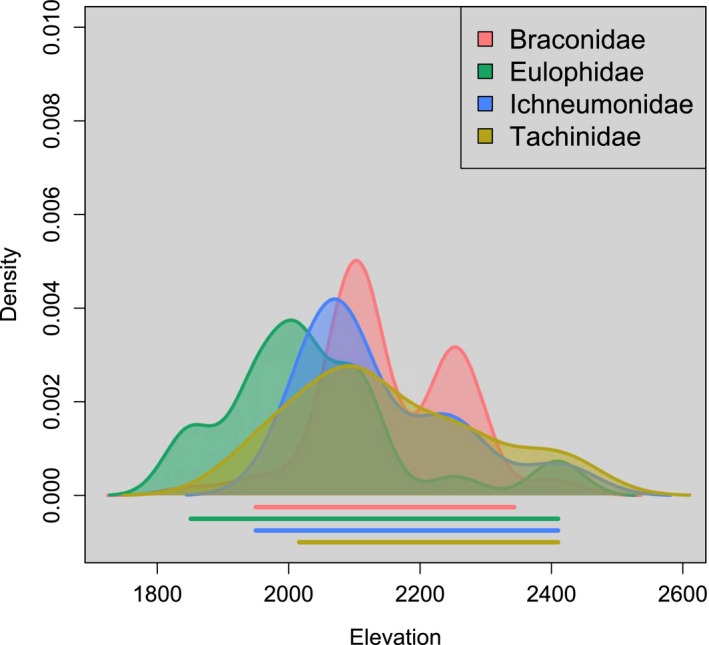
Parasitoid family density along elevation. Parasitoids reared and identified to family level included Braconidae, Eulophidae, Ichneumonidae and Tachinidae. Eulophidae had the highest densities at 2000 m and Braconidae, Ichneumonidae and Tachinidae had the highest densities at 2100 m. Lines under plots indicate 95% confidence intervals. This relationship is based on a linear model, while the path analysis includes residual variation from interacting variables.

For the structural equation models, the model that provided the best fit to the data is summarized by the path diagram in Fig. 2 (χ^2^ = 0.014; df = 1; *P* = 0.91; *P*‐values closer to 1 indicate a better fit to the data). There were several notable causal relationships supported by this model (Table 1; Figs 2b, S2). First, elevation had positive direct effects on phytochemical defense (standardized path coefficient (spc) = 0.10; slope (*B*) = 0.0009), *Eois* community diversity (spc = 0.11; *B* = 0.0004), and *Piper* community diversity (spc = 0.14; *B* = 0.0005). While Eulophidae parasitoids had the highest densities at 2000 m and Braconidae, Ichnuemonidae and Tachinidae parasitoids had the highest densities at 2100 m (Fig. 3), in our path model there is a linear relationship between elevation and parasitoid diversity while accounting for other factors that include *Piper* community diversity and high concentrations of phytochemistry (Fig. 2b; spc = 0.37; *B* = 0.0024). The effects of elevation on phytochemical defense also cascaded to the arthropod community; as phytochemical defense increased, *Eois* community diversity on *P. kelleyi* decreased (Fig. 2b; spc = −0.2; *B* = −0.12) and parasitoid diversity increased (spc = 0.26; *B* = 0.24) – adding to the strong direct effects of elevation on parasitoids (Fig. 2b). Increases in *Piper* plant community diversity also enhanced parasitoid diversity on individual *P. kelleyi* plants (spc = 0.11; *B* = 0.44) and had the strongest negative effect on *Eois* caterpillar diversity on individual *P. kelleyi* plants (Fig. 2b; spc = −0.48; *B* = −1.23). Finally, greater *Piper* plant community diversities are associated with decreased phytochemical defense in *P. kelleyi* (spc = −0.25; *B* = −1.04). Overall, the path model suggests that increases in phytochemical defense – specifically the prenylated benzoic acid – and *Piper* community diversity reduced *Eois* community diversity, while increasing parasitoid community diversity (Fig. 2b). Higher elevations are associated with greater *Piper*,* Eois* and parasitoid community diversities, as well as with greater *P. kelleyi* phytochemical defense.

### Caterpillar genetic variation, phytochemical variation, and elevation

**Figure 4:**
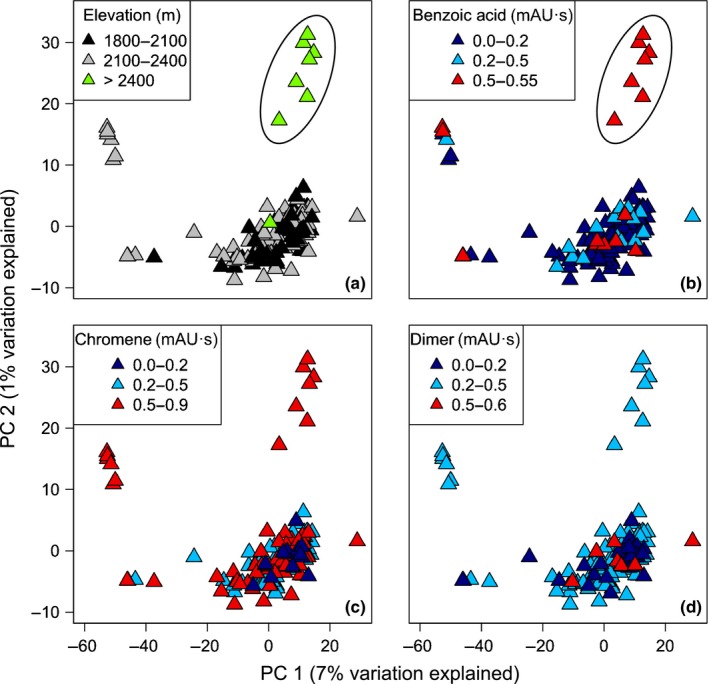
Principal components analysis illustrates genetic variation and structure across populations of *Eois encina* moths. Points represent genotypic data for 20 458 single nucleotide polymorphisms (SNPs) in each individual. The first two principal components (PCs) explained 7% (PC1) and 1% (PC2) of the genotypic variation across all individuals and loci and revealed previously undetected genetic differentiation in high‐elevation populations. Black circles denote individuals exhibiting genetic differentiation, which is correlated with high elevation and benzoic acid concentrations in plants. (a–d) Panels are the same principal components analysis, but differ in the overlaid gradient. Panel (a) illustrates an elevation gradient, with black being the lowest elevation and green being the highest elevation; (b–d) illustrate the prenylated benzoic acid, chromane and dimeric chromane concentration gradients, respectively. Dark blue is the lowest concentration, while red is the highest concentration.

After executing one lane of sequencing on the Illumina HiSeq platform, and conducting assemblies and variant calling as described in the Materials and Methods section, we retained genotypes at 20 458 SNPs in 155 *E. encina* individuals (accessible in Dryad, doi:10.5061/dryad.d67h6). The mean coverage per locus per individual was 1.25×. We used PC analysis to summarize patterns of genetic variation across all individuals. The first two PCs explained 7% (PC1) and 1% (PC2) of the genotypic variation, and suggest that *E. encina* from the highest elevation is genetically differentiated from that at lower elevations (Fig. 4a). There were other differentiated individuals, but we focus discussion on the highest elevation for which we have a hypothesis about phytochemical variation. The *P. kelleyi* plants that this population was occurring on had higher relative concentrations of the prenylated benzoic acid (Fig. 4b). Other potential patterns suggested by the PCA were not interpretable based on our original mechanistic hypotheses.

Multiple regression using distance matrices (Lichstein, 2007) was conducted to examine whether elevation, phytochemical variation and GPS location were predictors of genetic distances among *E. encina* individuals. Genetic variation was estimated using the PC scores of multi‐locus genotype likelihoods of *E. encina* individuals transformed into a distance matrix; three matrices were created based on all the PCs, PC1 and 2, and only PC2 scores. Table 2 depicts the results from multiple Mantel tests in which models differed based on the response variable: (A) all the PCs, (B) PC1 and 2, and (C) only PC2 scores. Increases in elevation and prenylated benzoic acid significantly explained *E. encina* genetic variation. Geographic location was only a significant predictor with PC2 score as the response variable.

**Table 2:** Results from multiple Mantel tests in which models differed based on the response variable

Model	Elevation	PBA	Chromene	Dimeric chromane	Location	Overall model
A – all PCs	pMc = 0.007; *P *=* *0.14	pMc = 16.89; *P *<* *0.001*	pMc = 3.74; *P *=* *0.54	pMc = −13.42; *P *=* *0.18	pMc = −1.69; *P *=* *0.79	*R* ^2^ = 0.05; *P *=* *0.001*
B – PC1 and PC2	pMc = 0.01; *P *=* *0.05*	pMc = 26.94; *P *<* *0.001*	pMc = −0.06; *P *=* *0.99	pMc = −12.64; *P *=* *0.38	pMc = −1.37; *P *=* *0.88	*R* ^2^ = 0.07; *P *<* *0.001*
C – only PC2	pMc = 0.02; *P *<* *0.001*	pMc = 20.21; *P *<* *0.001*	pMc = −3.54; *P *=* *0.31	pMc = −4.6; *P *=* *0.43	pMc = −9.82; *P *=* *0.01*	*R* ^2^ = 0.26; *P *<* *0.0001*

pMc, partial Mantel coefficient.

Predictor variables for all models were elevation, prenylated benzoic acid (PBA), chromene, dimeric chromane and GPS location. For the response variable, genetic variation was estimated using the principal component (PC) scores of multi‐locus genotype likelihoods of *E. encina* individuals transformed into distance matrices. Three matrices were created based on: A, all the PCs; B, PC1 and 2; C, only PC2 scores. Elevation and PBA were significant predictors of genetic variation in *Eois encina* populations.

*Predictor variables and overall models that were significant.

## Discussion

Intraspecific phytochemical variation is an underappreciated source of geographic variation in species interactions and their evolutionary outcomes (Bolnick *et al*., 2011; Zhang *et al*., 2015). At what scales might this variation shape community structure and generate divergent selection and population differentiation? We tested the hypothesis that phytochemical variation within a single species of host plant *P. kelleyi* drives community structure via subtle microhabitat differences in chemically mediated interactions between plants, their specialist herbivores and associated parasitoids. We also examined whether genetic structure across populations of specialist caterpillars was correlated with phytochemically variable microhabitats. We found evidence for substantial chemical variation among individual plants (Figs S3–S5; Tables S1, S2). The concentration of one important defensive compound, prenylated benzoic acid, increased with increasing elevation (Fig. S4). In turn, plant chemistry strongly predicted community structure at the herbivore and parasitoid trophic levels, with more toxic plants supporting a lower diversity of specialist herbivores, richer parasitoid communities, and higher levels of parasitism. Across populations of one of the specialist caterpillar species, our results suggest the potential for genetic differentiation between high‐elevation caterpillar populations and all others (Fig. 4), which could be mediated by high concentrations of plant secondary metabolites. Overall, the results are consistent with phytochemical variation shaping community structure, perhaps giving rise to geographic variation in the selection pressures experienced by specialist herbivores. Below, we discuss community structure and then evaluate possibilities for chemically mediated population structure.

Within our plots, associations between phytochemical defense and *Eois* assemblages on individual *P. kelleyi* plants were consistent with predictions that increased phytochemical defense causes decreased diversity of specialist caterpillars, while concurrently increasing parasitoid community diversity. Intraspecific phytochemical diversity had a negative influence on caterpillar diversity, consistent with previous studies demonstrating that increases in phytochemical variation within a host *Piper* species have negative effects on some *Eois* species but not others (Dyer *et al*., 2003, 2004; Richards *et al*., 2010, 2012). Interestingly, increased phytochemical diversity (measured as changes in relative abundance of the three defensive compounds) facilitated higher parasitoid diversity. This chemistry−parasitoid relationship could imply that increases in phytochemical variation of *P. kelleyi* may somehow attract more parasitoids for defense against caterpillars (Turlings & Ton, 2006; Wäschke *et al*., 2014) or that plant chemistry compromises the immune response of sequestering specialists (Smilanich *et al*., 2009) – the former is less likely as these compounds are not volatile. Could it be that volatile organic compounds are also increased and attracting richer parasitoid communities (e.g. Raguso, 2009)? It is worth pursuing this question, along with the interesting pattern of increased parasitism pressure, a result that contrasts with a previously reported trend that levels of parasitism decrease with increasing elevation in temperate zones (reviewed in Hodkinson, 2005).

*Piper* diversity within plots had the strongest negative effect on the diversity of *Eois* found on *P. kelleyi*, which is consistent with a hypothesis of associational resistance (Root, 1973; Barbosa *et al*., 2009). Alternatively, this negative relationship between *Piper* diversity and *Eois* diversity on our focal host plant could be a consequence of the highly specialized relationship these moths have with *Piper* combined with the fact that *Eois* moths are not very mobile. As a result, preferred hosts are likely to be less ‘apparent’ in high‐diversity *Piper* plots (Root, 1973; Barbosa *et al*., 2009). Higher diversity *Piper* communities also attracted more parasitoids that attack *Eois*. This result suggests that parasitoids are able to locate their preferred host even in the presence of complex cues from closely related nonhost plants (Erb *et al*., 2010; Wäschke *et al*., 2014). Alpha diversity of *Piper*,* Eois*, and associated parasitoids at a small scale (10‐m‐diameter plots) also increased with elevation, consistent with previous *Piper* diversity studies that demonstrated a strong effect of elevation on *Piper* and *Eois* diversity (without mid‐domain effects) in the eastern Andes (Brehm *et al*., 2007; Rodríguez‐Castañeda *et al*., 2010). Similarly, Rodríguez‐Castañeda *et al*. (2010) found that monophagous herbivore diversity increased with elevation, suggesting that colder temperatures and higher precipitation up the mountain are important for diversity by increasing specialization of herbivores and parasitoids on host plants. Our results contrast with studies in temperate systems. For example, Rasmann *et al*. (2014) found that Buprestid beetles tended to be more generalized and polyphagous at higher altitudes, and Pellissier *et al*. (2012) found that butterfly specialization decreased with increases in elevation; this was largely attributed to decreases in host plant abundance as elevation increased. As pointed out by Rodríguez‐Castañeda *et al*. (2016), there are not enough studies of chemically mediated trophic interactions across elevations to enable any generalizations to be made in tropical or temperate ecosystems, so it is not clear if the patterns reported here are part of a more general pattern of changes in plant chemistry and diversity.

Of the variables examined in our study, elevation was the main predictor of changes in phytochemical defense as well as the abundance of individual compounds. Concentrations of secondary metabolites were correlated with increasing elevation, which has been documented in a few studies with other classes of secondary metabolites, but there are exceptions (reviewed by Rodríguez‐Castañeda *et al*., 2016). Compounds for which there is a documented decline in concentrations of secondary metabolites with increasing elevation include terpenes (Hengxiao *et al*., 1999), alkaloids (Salmore & Hunter, 2001), and iridoid glycosides (Pellissier *et al*., 2012). As empirical data for changes in chemical defense across elevational gradients are still scarce (Rodríguez‐Castañeda *et al*., 2016), it is useful to examine the mechanisms that cause chemistry to change with elevation. In the case of *P. kelleyi*, the increase in defensive compounds at greater elevation could be attributable to the relationship between increased UVB radiation at higher elevations and the photoactive properties of the prenylated benzoic acid, chromene, and dimeric chromane (Figs S4, S5; Table S1, S2; Krause *et al*., 1999; Ruhland *et al*., 2013; Virjamo *et al*., 2014). UV light is known to affect concentrations of phytochemicals through a variety of mechanisms (Becker & Michl, 1966; Zangerl & Berenbaum, 1987; Downum *et al*., 1991; Padwa *et al*., 1996), and plants at higher elevations are exposed to a greater intensity of UV light compared with plants at lower elevations. The association between elevation and *P. kelleyi* phytochemistry was mostly attributable to changes in the relative concentration of prenylated benzoic acid in the leaves. Chromenes are particularly reactive in the presence of UV light (Becker & Michl, 1966; Padwa *et al*., 1996), and it is likely that increased UV radiation facilitates related biosynthetic links through oxidation of the prenylated benzoic acid and cyclization to the chromene, as well as through a photoinitiated dimerization to produce the chromane. The prenylated benzoic acid probably acts as the precursor, so an increase in one compound is usually associated with increases in the others, as we document here. Thus, *Piper* shrubs that produce phototoxic compounds could be more toxic at the top of elevational gradients in the Andes and less toxic towards the bottom, because of UV‐induced increases in abundance and diversity of secondary metabolites.

The possibility that changes in elevation and UV exposure modify the phytochemistry of *P. kelleyi* is relevant to the evidence for *Eois* population structure. We found evidence of subtle genetic differentiation for *E. encina* at the highest elevation (Fig. 4a), but little evidence for differentiation among other populations. The plants located at the highest elevation (> 2400 m) contained higher concentrations of prenylated benzoic acid (Figs S3–S5; Tables S1, S2), which could be a source of divergent selection. Local adaptation of *E. encina* to high‐elevation hosts with high prenylated benzoic acid concentrations could reduce gene flow by causing selection against migrants and low hybrid fitness (Zhang *et al*., 2015), which could explain the observed genetic differentiation among low‐ and high‐elevation caterpillars. One individual caterpillar was collected at a high‐elevation site that was not genetically grouped with the other highest elevation caterpillars (Fig. 4a). Interestingly, this one distinct high‐elevation caterpillar was feeding on plants containing a lower concentration of the prenylated benzoic acid (Fig. 4b), which could suggest that this individual was a migrant from lower elevation populations. We assume that the high‐elevation *E. encina* do not represent a cryptic species based on adult vouchers, genitalia dissections, and extensive photographic documentation of larvae. MRM models indicated that genetic distances were explained by chemistry while controlling for elevation (*R* ^2^ = 0.07; *P *<* *0.001). The genetic differentiation of *E. encina* collected at high elevation on high‐acid plants, although subtle, is consistent with the possibility of local adaptation limiting gene flow across these populations and with the hypothesis that phytochemical variation could generate geographically divergent selection across *E. encina* populations. Our results are similar to those of a recent study on the elm *Ulmus pumila L*., which demonstrated that leaf age is a source of divergence in two sympatric sister elm leaf beetles, *Pyrrhalta maculicollis* and *Pyrrhalta aenescens* (Zhang *et al*., 2015). Future studies with the *Piper* system should include a reciprocal rearing experiment comparing high and low populations of *Eois* to demonstrate that *Eois* populations at high elevation are adapting to higher concentrations of prenlyated benzoic acid in *P. kelleyi* plants.

Phytochemical variation could be an ecological source of natural selection on specialist herbivores. Such variation in plant defense has performance and fitness consequences for herbivores, and this is particularly true for specialist herbivores, which may be locally adapted to detoxify specific compounds (Dyer *et al*., 2003; Richards *et al*., 2010). The biological activity of the defensive compounds in *P. kelleyi* included decreased development rate, lower pupal mass and decreased survival when small amounts (i.e. 3.75% of the dry weight) were fed to naïve generalist caterpillars (Jeffrey *et al*., 2014). This amount is less than half of the 10% dry weight typically found in the leaves of *P. kelleyi* (Jeffrey *et al*., 2014). However, because this assay was performed on generalist caterpillars, it is unknown how specialist caterpillars respond to increased concentrations of the three compounds. Other studies have shown that *Piper* chemical defenses have subtle effects on *Eois* physiology rather than direct toxic effects (Dyer *et al*., 2004; Smilanich *et al*., 2009). For example, Richards *et al*. (2010) found that subtle changes in mixture diets (i.e. 0.4%) of *P. cenocladum* resulted in decreased survival rates and increased parasitism frequency in specialist *E. nympha* Schaus caterpillars. It is likely that at higher elevations the intensity of UV light is greater and can enhance the toxicity of these plants by increasing the abundance and evenness of defensive compounds, leading to selection against larvae that lack adequate physiological mechanisms for detoxification or tolerance (e.g. McCloud & Berenbaum, 1999).

### Conclusions

The results reported here represent a significant contribution to our understanding of the chemical processes maintaining biodiversity at different taxonomic and spatial scales. The study examined community and population structure at a fine scale by focusing on intraspecific host plant populations with distinct phytochemical profiles and associated herbivore communities. We found that variation in plant chemistry affected the caterpillar community (relative abundances of different caterpillar species) and genetic differentiation among populations of *E. encina*, the most common caterpillar species. The adaptive significance of phytochemical variation in response to UV light intensities, particularly whether it is plastic or genetic, as well as the potential role of phototoxicity have yet to be determined. Future studies are needed to examine whether the compounds are phototoxic and how this influences local adaptation and community assemblages of specialist and generalist consumers.

## Author contributions

A.E.G. wrote the first draft of the manuscript. L.A.D., M.L.F. and T.L.P. contributed substantially to revisions. A.E.G., L.A.D., M.L.F. and L.A.R. generated hypotheses and designed experiments. L.A.D., M.L.F., C.S.J. and A.M.S. funded experiments. A.E.G., L.A.D., T.R.W., C.R.M., W.S., L.A.S., E.J.T. and S.V‐C. collected field data. A.E.G., C.S.J., L.A.R., M.D.L. and C.D.D. conducted chemical analyses. A.E.G., M.L.F., J.P.J. and J.S.W. prepared the genomic library. T.L.P., C.C.N. and M.L.F. aligned and assembled genotypes. A.E.G., M.L.F., L.A.D., T.L.P., J.P.J. and L.A.R. conducted statistical analyses. E.J.T. identified *P*. *kelleyi* plant samples. J.S.M. conducted genitalia dissections to identify *Eois* species.

## Supporting information

Please note: Wiley Blackwell are not responsible for the content or functionality of any supporting information supplied by the authors. Any queries (other than missing material) should be directed to the *New Phytologist* Central Office.

**Fig. S1** A map illustrating the locations of *P. kelleyi* plant and caterpillar samples that were collected near Yanayacu Biological Station near Cosanga, Napo Province, Ecuador in the eastern Andes (00°36′S and 77°53′W).
**Fig. S2** Partial correlation plots from the structural equation model (Fig. 2a) for paths II, III, IV, V, VI and IX.
**Fig. S3** Principal component analysis examining the chemical similarity between individual *Piper kelleyi* plants using concentrations of prenylated benzoic acid, chromene and dimeric chromane of individual plants.
**Fig. S4** Linear regression examining the relationship between elevation and PC2 scores from the phytochemical PCA (Fig. S3).
**Fig. S5** Linear regression examining the relationship between elevation and PC1 scores from the phytochemical PCA (Fig. S3).
**Table S1** Variation explained by each component used in the principal components analysis
**Table S2** The first three eigenvalues of the correlation matrix for the principal component analysis, the proportion of total variance, and the cumulative variance for each principal component
