# Supplementary material for: Intraspecific phytochemical variation shapes community and population structure for specialist caterpillars
Source: New Phytol. 2016 Jun 9;212(1):208–19. doi: 10.1111/nph.14038 (PMC5089596; doi:10.1111/nph.14038)
Supplement: Supplementary file 1 — Fig. S1 A map illustrating the locations of P. kelleyi plant and caterpillar samples that were collected near Yanayacu Biological Station near Cosanga, Napo Province, Ecuador in the eastern Andes (00°36′S and 77°53′W). Fig. S2 Partial correlation plots from the structural equation model (Fig. 2a) for paths II, III, IV, V, VI and IX. Fig. S3 Principal component analysis examining the chemical similarity between individual Piper kelleyi plants using concentrations of prenylated benzoic acid, chromene and dimeric chromane of individual plants. Fig. S4 Linear regression examining the relationship between elevation and PC2 scores from the phytochemical PCA (Fig. S3). Fig. S5 Linear regression examining the relationship between elevation and PC1 scores from the phytochemical PCA (Fig. S3). Table S1 Variation explained by each component used in the principal components analysis Table S2 The first three eigenvalues of the correlation matrix for the principal component analysis, the proportion of total variance, and the cumulative variance for each principal component [file NPH-212-208-s001.pdf]

New Phytologist

***New Phytologist* Supporting Information**

Article title: **Intraspecific phytochemical variation shapes community and population structure for specialist caterpillars**

Authors: Andrea. E. Glassmire, Christopher S. Jeffrey, Matthew L. Forister, Thomas L. Parchman, Chris C. Nice, Joshua P. Jahner, Joseph S. Wilson, Thomas R. Walla, Lora A. Richards, Angela M. Smilanich, Michael D. Leonard, Colin R. Morrison, Wilmer Simbaña, Luis A. Salagaje, Craig D. Dodson, Jim S. Miller, Eric J. Tepe, Santiago Villamarin-Cortez and Lee A. Dyer

Article acceptance date: 26 April 2016

The following Supporting Information is available for this article:

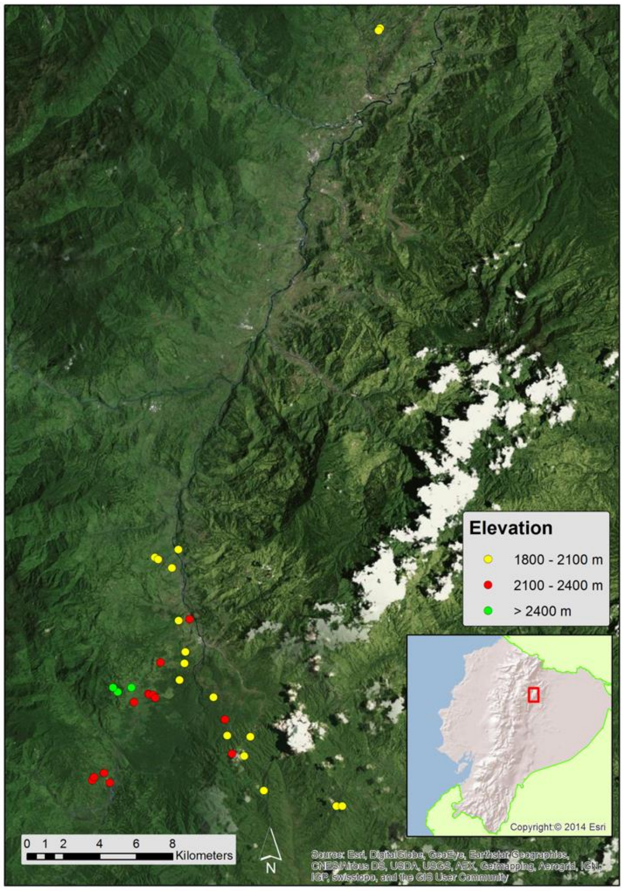

Fig. S1 Plant and caterpillar samples were collected near Yanayacu Biological Station near Cosanga, Napo Province, Ecuador on the Eastern Andes (00°36' S and 77°53' W). The red box on the inset map represents the general sampling location within Ecuador. This is a zoomed in view of the 32 plots within the red box. Plots are color coded based on elevation. The majority of sampling collections were made within 10 kilometers of one another. The yellow points north of Yanayacu were the farthest two sites.

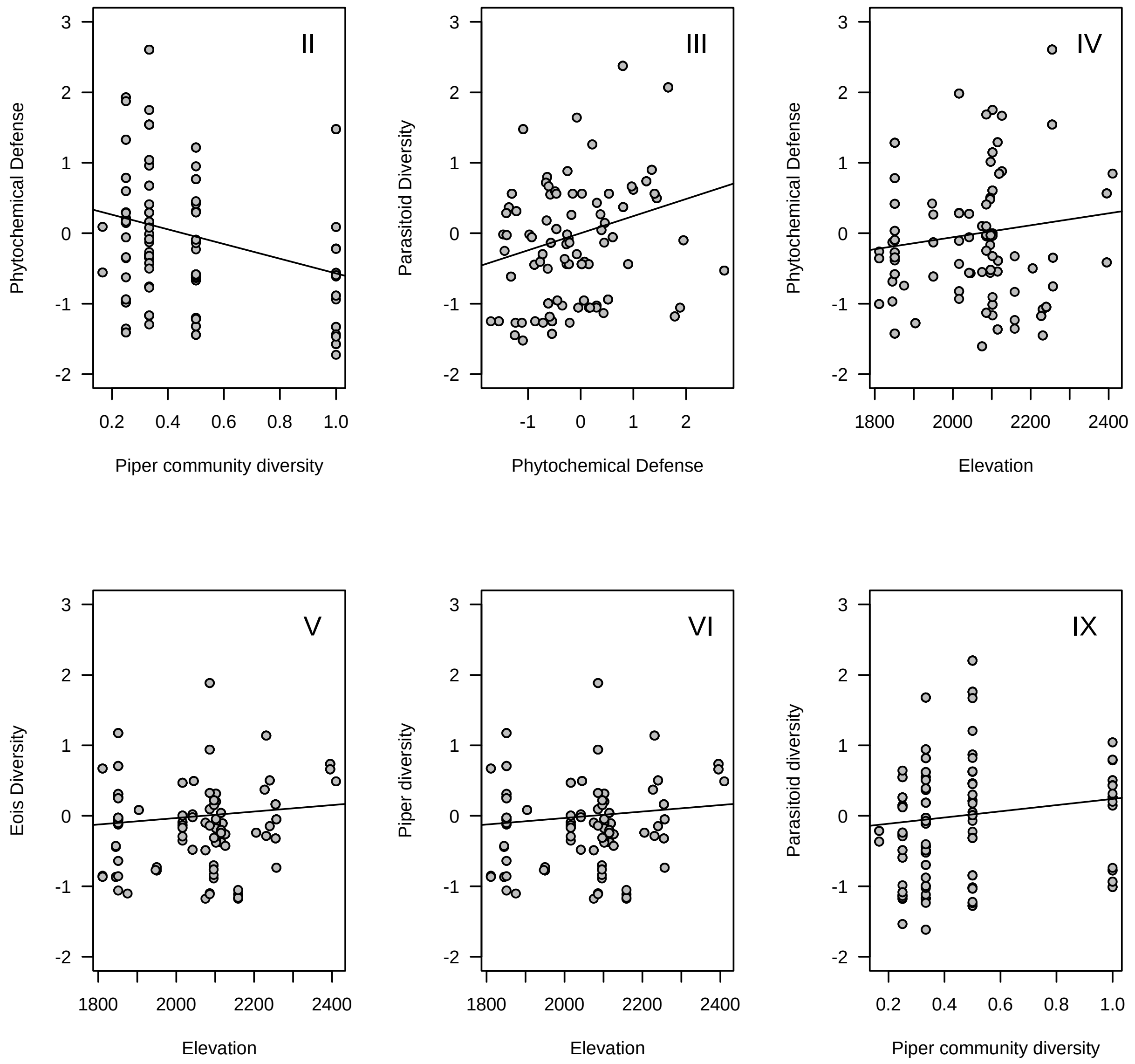

Fig. S2 Partial correlation plots from the structural equation model. Numbers in the top right correlate with the path number from Fig. 2.

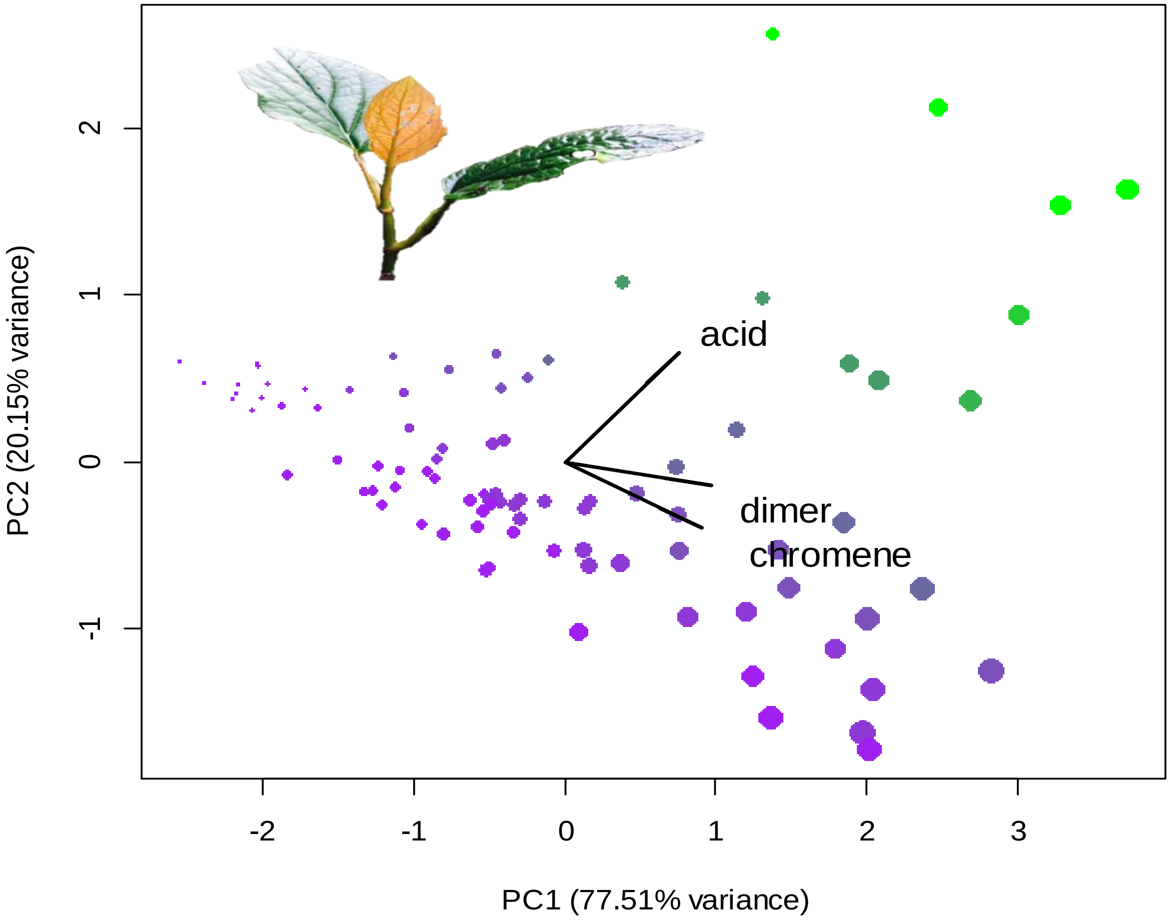

Fig. S3 Principal component analysis examining the chemical similarity between individual *Piper kelleyi* plants using concentrations of prenylated benzoic acid, chromene and dimeric chromane of each plant. Concentrations were obtained via HPLC spectroscopy with an internal standard. Each point represents an individual plant and clusters of points indicate overall chemical similarity. The light green color denotes plants having higher concentrations of benzoic acid, while the purple color denotes plants having low concentrations of this compound. The larger sized dots indicate the plants having higher concentrations of dimeric chromane and chromene. PC1 explained 77.51% of the variation, with the most variation explained by the chromene and the dimeric chromane. PC2 explained 20.15% of the variation, with the most variation explained by the prenylated benzoic acid.

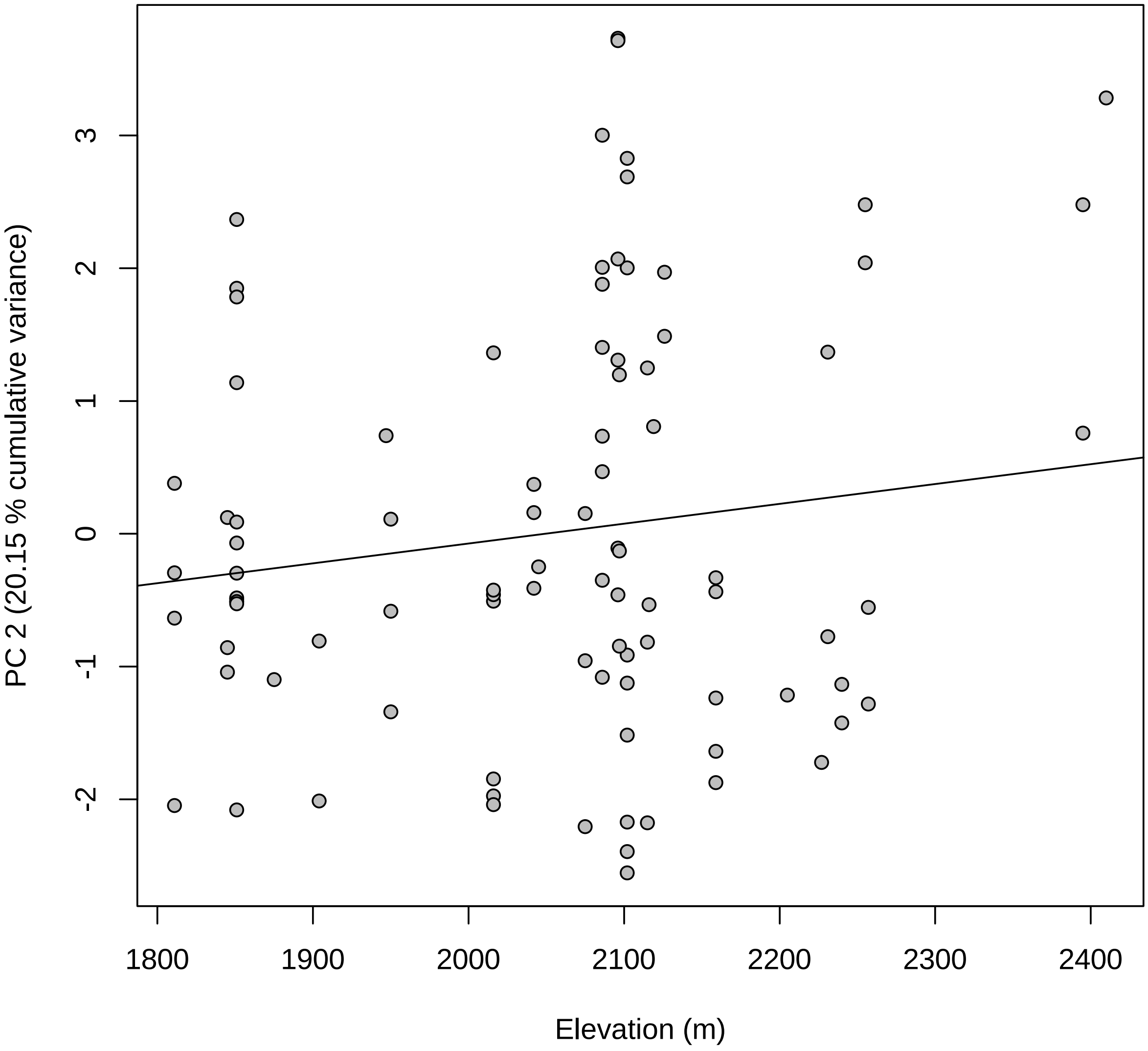

Fig. S4 Linear regression examining the relationship between elevation and PC2 scores from the phytochemical PCA. Most of the variation explained in PC2 is attributed to the prenylated benzoic acid. There is a significant increase in prenylated benzoic acid concentration as elevation increases (R^2= 0.08; P = 0.008; $F_{1,90}$ = 7.303).

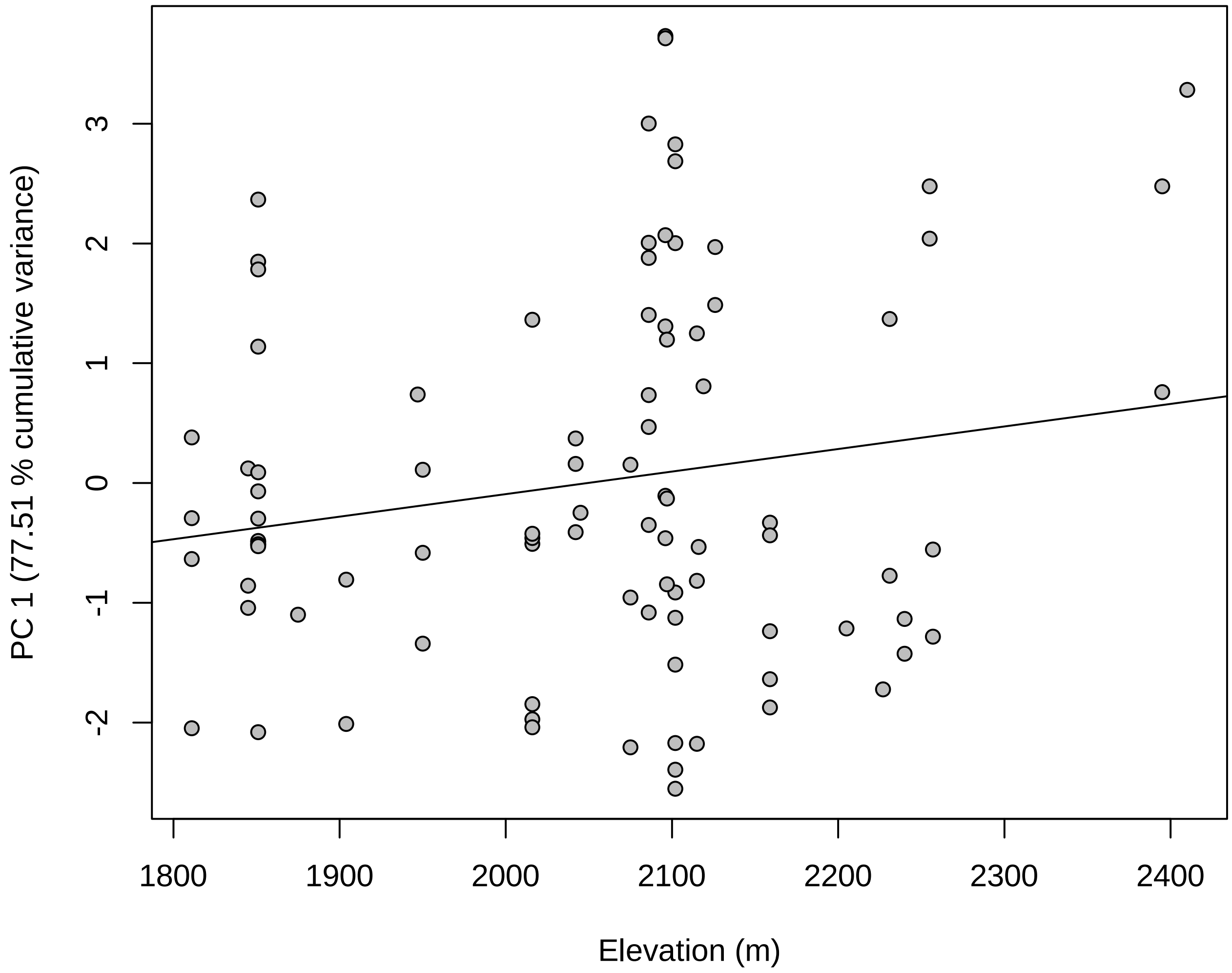

Fig. S5 Linear regression examining the relationship between elevation and PC1 scores from the phytochemical PCA. Most of the variation explained in PC1 is attributed to the chromene and the dimeric chromane. There was a slight increase in secondary metabolite concentrations as elevation increases (R^2= 0.03; P = 0.09; $F_{1,90}$ = 2.876).

Table S1 Variation explained by each component used in the principal components analysis. Chromene and dimeric chromane explained the most variation in PC1. Prenylated benzoic acid explained the most variation in PC2.

Variable	PC 1	PC 2	PC 3
Chromene	0.9	-0.4	0.16
Dimeric chromane	0.97	-0.13	-0.2
Prenylated benzoic acid	0.78	0.62	0.06

Table S2 The first three eigenvalues of the correlation matrix for the principal component analysis, the proportion of total variance, and the cumulative variance for each principal component.

Principal component	Eigenvalue	Proportion of total variance	Cumulative variance
1	2.33	77.51	77.51
2	0.6	20.15	97.66
3	0.07	2.34	100
